# Bridging Material Innovation and Environmental Safety: Aerogel-Based Magnetic Nanocomposites as Emerging Platforms for Water Decontamination

**DOI:** 10.3390/toxics14020115

**Published:** 2026-01-26

**Authors:** Elena-Theodora Moldoveanu, Adelina-Gabriela Niculescu, Denisa Alexandra Florea, Tony Hadibarata, Alexandru-Mihai Grumezescu, Dan-Eduard Mihaiescu

**Affiliations:** 1 Department of Science and Engineering of Oxide Materials and Nanomaterials, National University of Science and Technology POLITEHNICA Bucharest, 1–7 Polizu Street, 011061 Bucharest, Romania; elena.moldoveanu99@upb.ro (E.-T.M.); adelina.niculescu@upb.ro (A.-G.N.); denisa.florea@upb.ro (D.A.F.); tony.hadibarata@upb.ro (T.H.); 2 Research Institute of the University of Bucharest—ICUB, University of Bucharest, 90–92 Panduri, 050663 Bucharest, Romania; 3 Environmental Engineering Program, Faculty of Engineering and Science, Curtin University Malaysia, CDT 250, Miri 98009, Malaysia; 4 Department of Organic Chemistry, National University of Science and Technology POLITEHNICA Bucharest, 1–7 Polizu Street, 011061 Bucharest, Romania; danedmih@gmail.com

**Keywords:** aerogels, magnetic aerogels, aerogel-based nanoparticles, adsorption mechanisms, water remediation, ecotoxicological assessment, regeneration, recyclability

## Abstract

Currently, water pollution is one of the major global environmental sustainability and public health issues that requires efficient and viable remediation technologies, as existing decontamination methods face limitations. In this sense, this review aims to highlight the potential of multifunctional aerogel-based magnetic nanocomposites as a novel strategy for water decontamination by integrating magnetic nanostructures into aerogel matrices that promote high adsorption capacity, selective catalysis, and facile magnetic recovery. In this regard, providing a comprehensive analysis of their functional design, contaminant-removal mechanisms, and multifunctional performance is crucial for developing and optimizing a system capable of addressing complex pollutants through multiple mechanisms (e.g., adsorption, photocatalysis, and reductive pathways). However, ecotoxicological evaluations focus on the potential for nanoparticles to leach, induce oxidative stress, and cause aquatic toxicity, supporting the development of strategies that comply with safety principles. Additionally, this review examines the aerogels’ capabilities for regeneration, operational stability, and scalability across repeated-use cycles, as well as their potential for real-world wastewater applications. Moreover, future directions for these aerogels include the development of smart, stimuli-responsive aerogels, machine-learning-based modeling, and the use of green synthesis approaches to enable sustainable water remediation strategies.

## 1. Introduction

Water pollution remains a major global environmental crisis driven by urban expansion, population growth, rapid industrialization, and unsustainable resource use [1,2]. As a result, a complex mixture of pollutants, including pathogens, chemical waste products, heavy metals, pesticides, drugs, and compounds that pose a health hazard, can cause diseases and long-term health complications [1,2,3,4,5].

However, conventional water treatments, such as chemical precipitation, ion exchange, and electrochemical processes, are limited, require high energy consumption, and can pollute in turn. Consequently, there is an urgent need for more sustainable, efficient, and environmentally friendly treatment solutions, underscoring the need for advanced, multifunctional remediation materials [2,6].

Aerogels are promising materials for water remediation. Both simple and hybrid aerogels are the subject of research due to their remarkable properties, such as ultra-low density, high porosity, very large specific surface area, strong adsorption capacity, mechanical robustness, and tunable surface chemistry [6,7]. In the context of water remediation, this material can enable efficient pollutant removal while offering structural versatility. Improving their properties using magnetic nanoparticles can lead to the development of magnetic aerogels as an alternative to conventional strategies for contaminant removal, owing to their magnetic properties, catalytic activity, high porosity and adsorptive capacity, selectivity for contaminants, recovery and recyclability, and ease of synthesis (e.g., sol–gel) [6,8,9,10].

Although research on magnetic aerogels for water remediation continues to develop, there is a need for further work that integrates structural design, synthesis parameters, pollutant-removal mechanisms, and safety considerations. Current research typically studies adsorption, photocatalysis, magnetic separation, or ecotoxic effects separately, without correlating their interactions in complex wastewater environments. Therefore, this review aims to provide a broad perspective on the structural design, synthesis parameters, and architecture of aerogels, as well as their mechanisms and multifunctionality in removing complex pollutants from wastewater, to develop and optimize a system capable of addressing them. Nonetheless, this paper also presents ecotoxicological assessments, discussing the potential of nanoparticles (NPs) to leach, induce oxidative stress, and cause aquatic toxicity. This balanced approach supports the development of novel and efficient strategies that adhere to safety principles. In addition, this paper overviews the capabilities of aerogels for regeneration, operational stability, and scalability throughout repeated use cycles and real-world wastewater applications.

Furthermore, a future outlook for these aerogels may include the development of smart, stimulus-responsive aerogels, machine learning-based modeling, and the use of green synthesis approaches to enable sustainable water remediation strategies. Moreover, this work underscores the need for further research to better understand their importance and role in water decontamination. In this context, English language research articles and reviews were selected using information from scientific databases such as Web of Science, ScienceDirect, MDPI, Scopus, Frontiers, SpringerLink, and Wiley Online Library, using a variety of combinations among the following keywords: “aerogel-based nanocomposite”, “magnetic aerogels”, “water depollution”, “water remediation”, “magnetic separation”, “regeneration and recyclability”, “complex pollutants”.

Compared to several reviews that focus on synthesis routes of magnetic aerogels, while addressing water remediation through adsorption, photocatalysis, or magnetic separation independently, this review provides a unified and structured comparison of aerogel types and their performance on different pollutant classes, and dominant removal mechanisms, while explicitly integrating performance metrics, recyclability, and ecotoxicological constraints. By correlating material architecture with pollutant-specific removal pathways, this work offers a systems-level perspective that supports rational design of next-generation magnetic aerogels for real wastewater environments. In this sense, this work presents structural features that enable the creation of environmentally safe, multifunctional aerogel-based materials, providing a better perspective on magnetic aerogels as complex, reusable, and environmentally friendly remediation platforms.

## 2. Structural and Functional Design of Aerogel-Based Magnetic Nanocomposites

### 2.1. Correlation Between Physico-Chemical Properties and Synthesis Methods

Aerogel materials are well-known for their three-dimensional nanoporous structure and their excellent properties. Based on their chemical composition, aerogels can be classified into the following categories: organic (e.g., cellulose), inorganic (e.g., silica), carbon-based (e.g., carbon nanotubes, graphene oxide), and hybrid/composite aerogels [11,12,13,14]. From a structural standpoint, aerogels exhibit an open, three-dimensional network that yields extremely high porosity (80–99.8%), a very large specific surface area (typically 100–1600 m^2^/g), and ultralow densities (~0.003 g/cm^3^). These characteristics make aerogels ideal candidates for a wide range of applications, such as environmental remediation (e.g., heavy metal and dye removal from wastewater), medical uses (e.g., drug delivery systems, antibacterial, and disease detection), thermal insulation, sensors, catalysis, energy storage, and more [15,16,17]. However, they are inherently fragile, with weak mechanical properties, necessitating reinforcement strategies that include polymer crosslinking, fiber reinforcement, and the incorporation of nanomaterials to enhance their robustness without compromising porosity [16].

In this regard, various development and optimization approaches have been tackled to improve the properties of aerogels. As the synthesis route influences the morphology, porosity, and mechanical performance of aerogels, different fabrication strategies have been used (Table 1). The synthesis routes involve different parameters, such as precursor type and concentration, solvent type, catalyst and pH, surfactant content, and gelation temperature, each contributing to product characteristics. For instance, a lower precursor concentration makes the aerogel more fragile, while a higher concentration produces denser, stronger structures. Also, the solvent choice influences the porosity and homogeneity. In contrast, pH and catalysts modulate network crosslinking, along with surfactants that create the aerogels’ porosity [7,18].

In general, aerogels are obtained by a sol–gel process, in which a liquid “sol” representing the molecular precursor is transformed into a gel network by controlled hydrolysis, condensation, or assembly. This process is further followed by steps such as aging and drying to remove the solvent, while maintaining the aerogel’s structure [11,12,19]. In this context, each step in the aerogels synthesis offers different advantages and disadvantages that influence pore preservation, mechanical properties, and scalability. For instance, supercritical drying remains a favorable technique for aerogel drying, as it preserves fragile pore networks by removing solvent above its critical point, thereby avoiding capillary stress. This approach contrasts with freeze-drying, which promotes solvent elimination through sublimation [11,19]. However, despite the advantages of the sol–gel method, the obtained aerogels are amorphous, and post-synthesis calcination at high temperatures is needed to crystallize them and enhance the density of the pore network [20].

**Table 1:** Aerogel synthesis methods. Created based on the information from [17,21].

Synthesis Stage	Main Mechanism	Parameters	Advantages	Disadvantages
Sol–gel	Hydrolysis Condensation M-O-M Bond Formation	Temperature pH Solvent Type Precursor Concentration	Control of pore size, density, and homogeneity	Need for pH and water control during synthesis to prevent precipitation
Aging	Further step in the synthesis route: Continuous Condensation	Time Temperature Solvent Type	Provide improved mechanical properties Reduced defects	In excess, it causes shrinkage and densification
Drying	Supercritical Drying: The solvent is substituted, then brought above its critical point to prevent the formation of liquid–gas interfaces.	Solvent Type Temperature Pressure	Produce minimal shrinkage Provide good structural properties	Requires specialized high-pressure autoclaves.
	Freeze-Drying: The solvent freezing Vacuum sublimation	Freezing rate, solvent Material composition Material Viscosity	Simple setup Scalable	Ice crystals can reduce the porosity and surface area.
	Ambient Pressure: Solvent exchange Surface silanization	Surface modification Low-surface tension	Inexpensive Scalable	Risks of shrinkage

### 2.2. Depollution Mechanisms Using Aerogels

**Figure 1:**
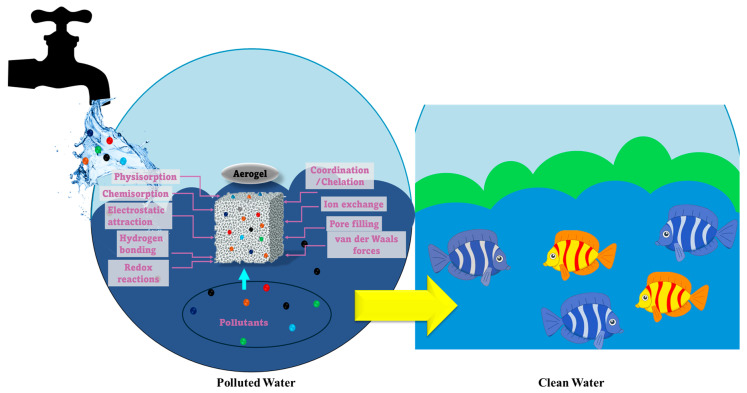
Mechanisms of pollutant removal. Created based on information from [25].

Aerogel applications in environmental remediation remain a promising strategy due to their capacity to remove persistent pollutants, such as pharmaceuticals, dyes, and oils, as well as heavy metals, radionuclides, and persistent organic contaminants, which are extremely difficult to remove efficiently by conventional treatments [22,23,24]. The efficiency of pollutant removal is determined by factors such as surface chemistry, pore size distribution, diffusion accessibility, and the ability to form strong interactions with contaminants, rather than simple physisorption, due to their high surface area and interconnected pore network [10,22,25]. Thus, pollutant removal by aerogels can occur through multiple and simultaneous mechanisms (Figure 1), with the process depending on both the pollutant and the aerogel chemistry [25].

Physical adsorption is driven by van der Waals forces and capillary action, and is enhanced by the hydrophobicity/hydrophilicity of the aerogel. In contrast, chemical adsorption implies electrostatic interactions, hydrogen bonding, and coordination with other functional groups (e.g., –OH, –COOH, –NH_2_, or thiol groups), allowing selective capture of specific pollutants [22,24,25,26]. Moreover, the functionalization of aerogels can enhance selectivity for particular pollutants, such as metal ions, dyes, and aromatic compounds [22]. In this regard, silica, cellulose, and carbon-based aerogels are excellent alternatives for removing oil and organic pollutants from water due to their surface wettability, which can be readily modified from hydrophilic to highly hydrophobic [9,27]. In this sense, hydrophobic aerogels are preferred for the absorption of immiscible oils/solvents, while hydrophilic ones are better suited for the removal of organic pollutants [27].

In addition, aerogels are used to capture heavy metals. For example, an aerogel based on nanofibrillated cellulose/silica functionalized with thiol can capture heavy metals such as Hg(II) due to the capacity of thiol–mercury complex formation, while aerogels based on polydopamine-coated multiwalled carbon nanotubes proved highly efficient for Cu(II) and Pb(II) removal through chelation and coordination involving N-, O-, and P-bearing groups [25]. Moreover, hexavalent chromium (Cr(VI)) removal is necessary because of its high toxicity, which stems from its strong oxidizability, high solubility, and dispersibility in water. However, Cr(VI) removal from water is hindered by the strong pH dependence of chromium speciation, the adsorbent surface charge, and the coupled Cr(VI) → Cr(III) reduction process [28]. In liquids, Cr(VI) can be presented as CrO_4_^2−^, HCrO_4_^−^, H_2_CrO_4_, and Cr_2_O_7_^2^ whose relative distribution varies with solution pH, determining their adsorption [28,29]. Due to its persistence and bioavailability, and the existing drawbacks in the adsorptive materials (e.g., deficient recovery, reuse, and incomplete detoxification), Zhao et al. [30] developed a cellulose/covalent organic framework (COF) composite aerogel (TBPM)to efficiently remove hexavalent chromium (Cr(VI)) from aqueous environments. In this sense, TBPM performed an excellent removal of Cr(VI), with rapid uptake, under acidic pH due to the protonation of surface amino groups and electrostatic interactions with negatively charged Cr(VI) species. Moreover, the aerogel maintained its adsorptive properties for Cr(VI) even in the presence of competing ions.

Other major pollutants include arsenate, phosphate, and cyanide. Arsenic is a well-known toxic substance, and its major species in water are arsenate and arsenite. Thus, Ye et al. [31] designed a magnetic konjac glucomannan (KGM) aerogel as an adsorbent for arsenite removal. Researchers observed that the aerogel exhibited pH-dependent adsorption of arsenite. Moreover, they observed that the presence of the Cl^−^, NO^3−^, and SO_4_^2−^ did not significantly hinder the adsorption of As(III). In contrast, the presence of SiO_3_^2−^ and PO_4_^3−^ presented the opposite effect in As(III) adsorption. Also, Rahman et al. [32] performed their studies on magnetic cellulose nanofibril aerogel CNF–IONP for arsenic removal. Compared with the previous study, CNF–IONP exhibited high adsorptive performance for both As(III) and As(V) in water at acidic to neutral pH (3–7), with rapid uptake of these two compounds. However, further research is needed to see the efficiency in the case of competitive adsorption. Phosphate is predominantly found in nature and is not especially toxic, but, in excess, it can lead to serious water pollution by stimulating algal blooms and eutrophication, which contribute to the deterioration of natural water ecosystems [33]. Thus, studies started to focus on obtaining adsorptive materials for phosphate decontamination. An example is the aerogel reported by Amaly et al. [34], who developed an alginate-based composite aerogel, MT^+^@QAlg, for the adsorption and recovery of phosphate and nitrate from livestock wastewater. Researchers obtained a composite with rapid, efficient removal of both phosphate and nitrate across a broad pH range (3–9) and maintained high removal efficiency even at high ionic strength. However, competition among anions (e.g., sulfate, chloride, and carbonate) resulted in only a slight decrease in aerogel performance in phosphate and nitrate selectivity.

Their structural characteristics influence the efficiency of aerogels in removing pollutants. In this regard, pore distribution, surface charge, cross-linking density, and morphology contribute more to efficiency than specific surface area. Thus, pore distribution and size control accessibility and transport as follows: micropores contribute to the formation of strong adsorption potentials and favor the adsorption of small molecules (e.g., metal ions). At the same time, mesopores promote rapid diffusion and facilitate adsorption for dyes and organic pollutants. In addition, macropores are preferred for oil and solvent adsorption because they reduce the specific surface area and minimize mass transfer by improving adsorption. [22,34,35]. Furthermore, surface charge, together with chemical heterogeneity, favors selectivity and low-pressure performance by promoting electrostatic interactions, chelation, and π–π interactions, thereby forming functional sites that facilitate the efficient adsorption of contaminants [22,34,35].

In addition, cross-linking is another important parameter, as it helps balance the mechanical stability of aerogels and can also influence the adsorptive properties of the materials. Thus, excessive cross-linking can lead to diffusion restriction, while insufficient cross-linking compromises the pore structure and reusability of aerogels [22,35].

In this sense, aerogels with moderate surface area and optimized hierarchical porosity, accessible load, and structural resilience have the potential to be used in practical wastewater remediation scenarios.

Due to their complex structure, formed by interconnected pores and hierarchical networks, the kinetics of absorption must be monitored to determine whether the process is governed by surface reactions or by internal mass transfer through the pores. This aspect is critical and is described in a study conducted by Sen Gupta and Bhattacharyya [36]. In this sense, they demonstrated that the pseudo-second-order (PSO) and intraparticle diffusion (IPD) models represent different, complementary aspects of adsorption kinetics. Thus, the PSO model is used to identify surface-reaction control arising from adsorbate–adsorbent interactions during chemisorption, while the IPD model provides insights into mass-transfer limitations within porous materials. In their research, the authors concluded that diffusion can influence but not limit adsorption, which means that several kinetic models should be considered together.

Regarding aerogels, Kurmysheva et al. [37] investigated the adsorptive capacities of graphene oxide aerogels (GOA) and reduced graphene oxide aerogels (rGOA) for organic pollutants such as 2,4-dichlorophenoxyacetic acid and salicylic acid. In this sense, the surface chemistry of GOA, which has a higher surface area and is rich in oxygen-containing functional groups, exhibited significantly higher adsorptive capacity and faster kinetics compared to rGOA. Moreover, the kinetic analysis provided valuable information: the PSO model had the best fit, compared to IPD, which indicates a multi-step process, implying surface adsorption followed by pore diffusion rather than diffusion-limited control. Similarly, Nguyen et al. [38] developed a cellulose nanofiber/graphene oxide (CNF/GO) composite aerogel with high porosity and robust mechanical properties. This aerogel promoted rapid, highly efficient dye adsorption. PSO kinetics provide an excellent fit to the researcher’s experimental data. Thus, the results show that surface-controlled chemisorption dominates the overall rate, while electrostatic attraction and π–π interactions govern the adsorption mechanism. Also, a lack of diffusion-limited behavior was observed, which further suggests that internal mass transfer can improve but not affect the adsorption rate. Moreover, Bober et al. [39] report consistent conclusions. In this regard, they developed a three-dimensional polypyrrole aerogel used for hexavalent chromium removal. PSO kinetics demonstrated that the chemisorption is driven by electrostatic attraction and anion exchange, governing the entire adsorption rate. Moreover, the IPD model reveals a multi-stage adsorption behavior, indicating that intraparticle diffusion contributes, but does not significantly influence the adsorption kinetics.

Overall, it is confirmed that pseudo-second-order kinetics and intra-particle diffusion analysis are complementary in observing adsorption processes in aerogel-based systems. Thus, PSO can provide information on surface reactions, which, in the presented studies, were observed to be the dominant rate-controlling step. In addition, IPD modeling provides insight into how pore structure and internal mass transport influence adsorption, allowing observation of mechanical behavior during adsorption in advanced porous materials such as aerogels.

Furthermore, pollutant adsorption can be influenced by factors such as pH, temperature, and ionic strength. Specifically, pH can influence the surface charge, ionization degree, and pollutant removal efficiency; temperature affects reaction rates; and ionic strength influences adsorption by altering adsorbent/adsorbate parameters [26,40].

Despite their advantages, magnetic aerogel’s performance in pollutant removal can be intrinsically influenced by their kinetics, surface chemistry, competitive and structural limitations. In this regard, adsorption kinetics can be governed by the balance between the speed of surface reactions and resistance to mass transfer. Thus, magnetic materials initially exhibit faster absorption, owing to the numerous accessible active sites. However, diffusion resistance and progressive saturation occur, leading to a slowdown in adsorption capacity as equilibrium approaches [41,42]. These drawbacks arise from the high tendency of NPs to agglomerate, pore blockage, and uneven magnetic loading. These findings were demonstrated by Shi et al. [42] and Nguyen et al. [43]. In this sense, comparing the use of magnetic fibers with magnetic aerogels, an improvement in diffusion pathways is observed, with open pathways that maintain interconnected porosity, thereby improving intrinsic adsorption kinetics under practical conditions. Moreover, surface chemistry can control adsorption capacity via reactive hydroxyl groups, iron–oxygen coordination sites, and oxygen-containing functional groups [41,42,43]. Also, adsorption is strongly influenced by the pH. Thus, at low pH, protonation can prevent metal binding due to electrostatic repulsion, whereas at higher pH, deprotonation promotes metal binding [41,42]. It was observed that the optimal adsorption can occur under mild acidic conditions [43].

At the same time, competitive adsorption is a major drawback in pollutant removal. Thus, in multi-component systems, metal ions, pollutants, and hydrolyzed species compete for a limited number of surface binding sites. In this sense, this competition reduces the capacity of pollutant adsorption and selectivity. For example, in heavy-metal adsorption, hydroxide ions at alkaline pH directly compete for surface sites, thereby significantly decreasing adsorption. Similarly, in nanocellulose-based aerogels, competition can be observed when the water contains inorganic ions or organic matter. Thus, these interactions must be considered when transitioning from laboratory applications to actual wastewater treatment [41,42,43,44,45]. Moreover, the structural characteristics of aerogels can govern the adsorption capacity, kinetics, magnetic separability, and recyclability. High surface area, interconnected porosity, uniform dispersion of magnetic nanoparticles, and mechanical robustness can improve adsorptive properties. In contrast, structural degradation such as pore collapse, particle clustering, or framework densification significantly impairs performance [41,42,43,44,45].

Beyond kinetic considerations, adsorption equilibrium and thermodynamic analyses provide essential insight into the nature and feasibility of aerogel–pollutant interactions. In this regard, adsorption isotherm modeling and thermodynamic analysis contribute to understanding the mechanisms of interaction between adsorbents and pollutants. Isotherm models (such as Langmuir, Freundlich, or Temkin) can help determine the distribution of the adsorbate at equilibrium and provide information for determining equilibrium constants. Thus, the thermodynamic parameters of adsorption (ΔG°—the change in standard Gibbs free energy of adsorption, ΔH°—standard enthalpy change in adsorption, and ΔS°—standard entropy change in adsorption) are evaluated when the equilibrium constant is derived from isotherms and expressed in a dimensionless form, respecting the principles of chemical equilibrium [46]. This assessment is essential for porous materials such as aerogels, as these parameters provide information on the spontaneity of the process, the energetic nature of the interactions, and changes in order at the solid–solution interface [47]. Table 2 presents a comparative analysis of aerogels used to remove pollutants, such as metal ions and organic dyes, from water. This analysis aims to highlight the adsorption capacity of contaminants, the predominant kinetic models and isotherms, the thermodynamic parameters, and the time to reach equilibrium. Thus, graphene-based and doped silica aerogels exhibit high adsorption capacity for dyes, whereas hybrid or magnetic aerogels are effective for removing metal ions. In this sense, the analysis demonstrates the possibility of using these materials in real wastewater treatment applications.

**Table 2:** Comparison of adsorption capacities and kinetic/isothermal models for aerogels used in water purification.

Aerogel Adsorbent	Pollutants	Max. Adsorption Capacity (mg/g)	Kinetic Model	Isotherm Model	Thermodynamics	Equilibrium Time	Ref.
Magnetic MnFe_2_O_4_—cellulose aerogel	Cu(II)	72	PSO	Langmuir	Spontaneous, chemisorption-dominated	~100 min	[48]
Graphene oxide aerogel (GOA)	MB	416.7	PSO	Langmuir	ΔG° < 0 (spontaneous), ΔH° > 0 (endothermic), ΔS° > 0	~120 min (30 min/4 days)	[49]
GOA	2,4-D, SA	SA: 57.61 2,4-D: 42.63	PSO	Freundlich	Chemisorption-dominated	~150 min	[37]
Hydrophilic silica aerogel (HPSA)	CV	137.17	PSO	Temkin	ΔG° < 0, ΔH° = −6.49 kJ/mol (exothermic)	~120 min	[50]
Titania-doped silica aerogel (TdS)	MB, CV	CV: 159.89 MB: 131.59	PSO	Langmuir	Predominantly physisorption	~240 min	[51]
Chitosan–silica hybrid aerogel	Cd^2+^, Ni^2+^	Cd^2+^: ~58 Ni^2+^: ~59	PSO	Langmuir	ΔG° < 0, ΔH° < 0, physisorption	60–180 min	[52]
Cellulose aerogel (paper waste)	CR, MB, RhB, NGB	CR: 14.48 MB: 13.54	PSO	Langmuir & Freundlich	Physical adsorption	~60 min	[53]
Graphene/La(OH)_3_ Aerogel (GLA)	Phosphate (PO_4_^3−^)	GLA-2: 22.21 GLA-6: 54.55 GLA-10: 76.85	PSO	Langmuir & Freundlich	Not reported	~24 h	[54]
Lanthanum-based aerogel beads (LCM)	PO_4_^3−^	77.49	PSO	Freundlich	Endothermic adsorption	~360 min	[55]
nZVI@NCA	Cr(VI)	nZVI@NCA600: 178.72 nZVI@NCA900: 298	PSO	Sips	Spontaneous (ΔG < 0), endothermic (ΔH > 0), ΔS > 0	~300 min	[56]

Recent studies have demonstrated that adsorption alone is not sufficient for efficient pollutant removal, due to the high risk of inefficient removal and secondary pollution. Thus, aerogels are limited in large-scale applicability, and interest in integrating adsorption with complementary catalytic pathways is increasing [57]. However, the adsorptive properties of pure aerogels regarding contaminants need improvement due to their weak physical adsorption of pollutants [58].

### 2.3. Enhancement of Aerogels in Pollutant Removal Using Magnetic Nanoparticles

**Figure 2:**
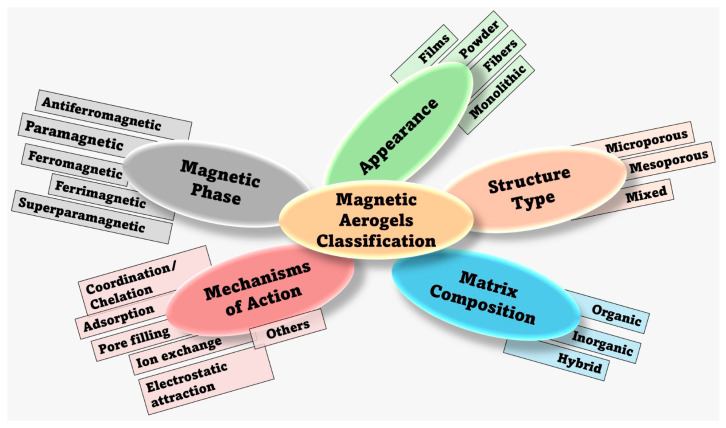
The classification of magnetic aerogels. Created based on the information from [21,25,26,59,60,61,62].

To obtain composites with enhanced adsorbent properties, using NPs can be considered a viable option. Thus, aerogels can serve as versatile hosts for NPs, enhancing their properties and enabling the creation of new composites with electronic, magnetic, and mechanical properties. Hence, various magnetic aerogels have been developed, being classified as shown in Figure 2.

Magnetic nanoparticles (MNPs) such as magnetic oxides (e.g., iron oxide) [63] and metallic NPs (e.g., nickel, cobalt) [64,65] can be incorporated into the aerogel matrix to obtain magnetically recoverable and reusable composites with applications in various fields, especially environmental remediation and water treatment [8,62,66]. In this sense, these composites can be manipulated by the magnetic field and promote the separation of contaminants [41,67]. Originally, the sol–gel method was developed for silica and then extended to carbon-based aerogels. It has been improved to synthesize a variety of inorganic aerogels, including oxides, chalcogenides, nitrides, carbides, and fluorides [20]. Typically, magnetic aerogels are obtained by dispersing MNPs in a precursor solution, and then following the previously mentioned steps to get a 3D composite with a large surface area and field-responsive behavior [66].

Among these systems, the most widely used are magnetite (Fe_3_O_4_) based nanostructures [68]. Their mixed-valence iron oxide, featuring Fe^2+^ and Fe^3+^ at octahedral sites, enables rapid electron transfer and underlies their notable electrical, catalytic, and magnetic properties [68,69]. On this point, their magnetic behavior enhances their recyclability and, in water depollution, Fe_3_O_4_ catalysts or sorbents can be recovered after application of an external magnetic field, minimizing secondary pollution and enabling reuse over multiple treatment cycles [24,69]. Adsorption interactions of magnetic-based nanoadsorbents are characterized by several types of adsorption interactions, which are specific to each type of pollutant and the MNPs’ properties (e.g., high surface area, surface hydroxyl groups, and tunable surface charge) [67,70]. For instance, several studies have demonstrated the capacity of magnetic aerogels for water decontamination applications. Li et al. [71] proposed a Fe_3_O_4_@TiO_2_/SiO_2_ aerogel that demonstrated rapid magnetic separation, high structural stability, and high adsorptive capacity even after multiple reutilizations. Similarly, Yuan et al. [72] developed a cellulose nanofiber magnetic aerogel (Fe_3_O_4_/CNF/PEI/SHMMT) with strong superparamagnetic responsiveness, demonstrating improved mechanical robustness and high adsorption capacity, confirming that Fe_3_O_4_ significantly enhances both recyclability and operational durability of aerogels. Moreover, Cheng et al. [41] designed a hybrid aerogel, Fe_3_O_4_@CTS-BDAT, that demonstrates its efficiency through dipole–dipole interactions, enabling faster magnetic capture and high separation efficiency. In this regard, Fe_3_O_4_ integration into aerogels indicates accelerated separation kinetics and sustained long-term functionality.

**Figure 3:**
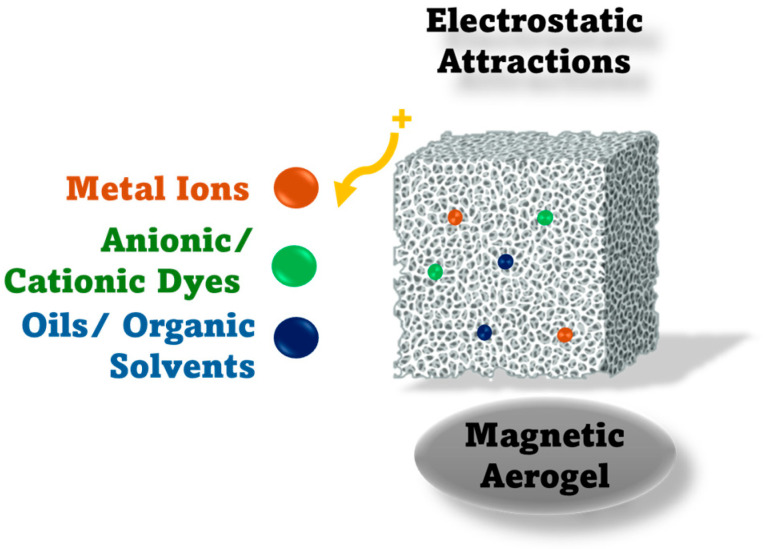
Representation of electrostatic attraction between magnetic aerogel and pollutants. Created based on information from [67,73,75].

Thus, composites based on MNPs can exert electrostatic attraction to aromatic dyes and pesticides (e.g., methyl orange, acid orange, and triazole fungicides) (Figure 3). At the same time, π–π stacking and hydrophobic interactions occur due to the aromatic structures of pollutants and the surface functionalization of MNPs (e.g., graphene oxide) or aromatic polymers, promoting their surface adhesion to MNPs [67,73,74].

Yet, the ferrite catalysts can simultaneously degrade pollutant molecules via Fenton-like reactions. Cationic metals such as Pb^2+^, Cu^2+^, and Ni^2+^ can interact with negatively charged oxygen sites (e.g., Fe–O^−^ sites) of MNPs, promoting coordination and inner-sphere formation [76]. Moreover, the adsorption kinetics of MNPs-based composites (e.g., Fe_3_O_4_-based composites) typically follow a pseudo-second-order or Langmuir isotherm model, suggesting that chemisorption is governed by the formation of surface complexes, which are improved by the electrostatic attraction between adsorbent surfaces and ionic species in solution [77]. Based on these principles of adsorption, recent advances in this field have begun to integrate MNPs (e.g., Fe_3_O_4_) with biopolymers (e.g., amphiprotic cellulose) and carbon-based frameworks (e.g., graphene oxide), which improve the stability, recyclability, and functionality of hybrid magnetic aerogels [78]. In this context, Xiong et al. [78] obtained a three-dimensional porous network with multiple both cationic and anionic active sites that promote simultaneous adsorption of oppositely charged pollutants via combined electrostatic interactions, hydrogen bonding, and π–π stacking mechanisms. The study by Li et al. [79] also demonstrated the same absorption mechanisms of aerogels toward various contaminants, including organic dyes (methylene blue, methyl orange), heavy metal ions (Pb^2+^, Cd^2+^, Cr^6+^), and organic solvents. Moreover, researchers demonstrated aerogel’s rapid adsorption rates, reutilisabiltity, and stable structure over multiple cycles with minimal loss in efficiency.

**Figure 4:**
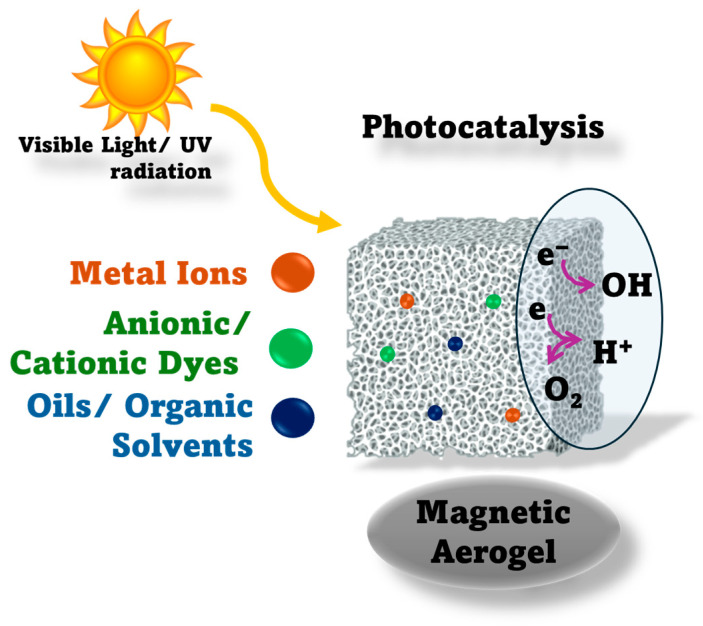
Representation of photocatalytic pollutant removal processes using magnetic aerogels. Created based on information from [83,84].

Beyond adsorption-based mechanisms, photocatalysis represents another strategy for pollutant removal (Figure 4). In this regard, this approach relies on semiconductor nanomaterials such as TiO_2_, ZnO, Fe_2_O_3_, FeTiO_3_, Al_2_O_3_, and Cu_2_O, which can generate electron-hole pairs under UV/visible light. These carriers generate redox processes resulting in reactive oxygen species (ROS) such as holes (h^+^), free electrons (e^−^), oxygen radicals (O_2_^−^), hydrogen peroxide (H_2_O_2_), and hydroxyl radicals (OH), which can degrade the aromatic rings and halogen substituents in pollutants’ chemical structure, promoting dehalogenation, ring opening, and mineralization to CO_2_ and H_2_O [24,80,81,82]. In this sense, coupling aerogels with these NPs can enable simultaneous adsorption and catalytic degradation of pollutants (e.g., dyes) under light, combining physical capture with in situ destruction of organic pollutants [23].

Sun et al. [85] developed a TiO_2_ aerogel modified with polyvinyl alcohol (PVA) to modify pore size. Researchers observed that the pore size influences the aerogel’s photocatalytic properties. This aerogel has a high degradation rate for HCHO under UV light. Electron–hole pairs (e^−^/h^+^) generate reactive oxygen species (O_2_^−^, OH), which oxidize HCHO into CO_2_ and H_2_O. Gallegos-Cerda et al. [86] present a cellulose, carbon nanotubes, and TiO_2_ NPs based aerogel for photocatalytic removal of organic dyes from wastewater. Thus, the experiments have been run on rhodamine B (RB) and methylene blue (MB), which showed high degradation rates under UV light within 110 min. Another study [66] developed a tri-functional S-scheme aerogel, providing insights into how its 3D architecture improved the charge separation, dye degradation, and efficient antibiotic removal under solar irradiation. Another work by Kong et al. [87] engineered a microporous conjugated polycarbazole (CPOP) –based aerogel that improved visible-light absorption, as demonstrated by Rhodamine B adsorption, and preserved catalytic activity after repeated reuse. In addition, Yao et al. [88] indicate the capacity of Ag/Fe@LCG aerogels to integrate adsorption with photo-Fenton-like degradation, with high adsorption of tetracycline and bisphenol A within 90 min, supported by accelerated Fe(II)/Fe(III) cycling and plasmonic enhancement.

However, photocatalytic degradation involves only oxidative pathways driven by ROS. In this case, reductive strategies have also been studied for the removal of persistent pollutants. Thus, reductive dechlorination frequently involves nanoscale zero-valent iron (nZVI) or bimetallic particles such as Fe/Pd and Fe/Ni [24,89,90]. This mechanism occurs via electron transfer, replacing chlorine atoms in pollutants with hydrogen, promoting efficient detoxification and improving the biodegradability of persistent pollutants [24].

For example, Wang et al. [91] developed a biomass-derived carbon aerogel for uranium removal and reduction from wastewater. Thus, researchers synthesized carbon-encapsulated nano zero-valent iron (nZVI) particles incorporated into biomass-derived carbon aerogel. The tests performed on this composite indicated that it achieved an efficiency of ~90% within 60 min and a maximum adsorption capacity of 720.8 mg/g, thereby overcoming the limitations of traditional iron-based adsorbents. Moreover, researchers studied the mechanisms involved in the removal process. In this sense, a coupled adsorption–reduction process was observed, in which U(VI) ions were firstly absorbed due to electrostatic interactions and coordination with oxygen-containing functional groups, followed by U(VI) reduction to insoluble U(VI) due to the electron transfer from Fe^0^ and Fe^2+^ species within the composite. Still, recent research continues to investigate the concept of bio-hybrid systems targeting organic pollutants. Zhou et al. [92] developed a novel aerogel that marks the transition from effectively combined carbon encapsulation and redox reactivity to nZVI@UiO-66-NH_2_/TCNF for pollutants removal, integrating the metal–organic frameworks (MOFs), nanocellulose, and nZVI, providing a stable aerogel, with electron transfer capacity and selectivity enabling an efficient reduction of chlorinated nitroaromatic compounds (e.g., p-chloronitrobenzene (p-CNB)). This aerogel achieved 85% degradation of p-CNB within 3 h, with a high rate of removal and selectivity compared to 4-chloroaniline (p-CAN) after 24 h. The high performance of NZVI@UiO-66-NH_2_/TCNF is attributed to its enhanced π–π interactions and electrostatic attraction between –NH_2_ and –NO_2_ groups, and it remains stable under various environmental conditions. Moreover, a synergistic adsorption-reduction process was observed in p-CNB adsorbed due to π–π stacking and Lewis acid–base interactions, followed by the reducing activity of nZVI, which creates active sites through Fe–O–Zr bridges to form p-CAN. This composite material has increased potential for efficient, selective pollutant removal in complex aqueous environments. Zeng et al. [93] further demonstrated that heavy metals and dyes are efficiently immobilized through coupled radical and non-radical pathways using magnetic bio-based aerogels, achieving efficient complexation and catalytic degradation of pollutants.

Another potential in the development of advanced magnetic aerogels may lie in the use of porphyrin photocatalysts. This compound is being studied, along with various sorbents, for its potential to decontaminate water of pollutants (e.g., metal ions, organic dyes, and antibiotics) [94,95,96]. First, Quadrado et al. [96] developed a hybrid aerogel based on poly(acrylic acid) (PAAc) and poly(vinyl alcohol) (PVA) to serve as a base for supporting photoactive porphyrin catalysis. The researchers obtained three types of aerogels containing different porphyrins, namely a free-base porphyrin (TMPyP), a zinc metalloporphyrin (TMPyPZn), and a manganese metalloporphyrin (TMPyPMn). These aerogels aim to degrade organic pollutants such as amoxicillin, caffeine, and naproxen by producing ROS. The study showed that the aerogel containing TMPyPMn has the best efficiency. The Mn-porphyrin is particularly effective at generating •OH radicals, both directly and through secondary reactions involving superoxide species and transient Mn(IV) intermediates. Sun et al. [97], on the other hand, have developed a multifunctional material based on Fe_3_O_4_@SiO_2_ core/shell onto which a porphyrin-based fluorescent receptor is chemically immobilized, to simultaneously detect, adsorb, and remove mercury(II) ions (Hg^2+^) from water. Remarkably, the material exhibits high selectivity toward Hg^2+^, demonstrating an excellent pollutant removal capacity. Moreover, the study showed the efficient separation of the adsorbent from the solution using a magnetic field. Thus, the development of aerogels with such potential merits requires further research integrating selective optical sensing, high-efficiency adsorption, and magnetic separability into a single material.

Notwithstanding the advantages offered by the integration of NPs into aerogel composition, such as a highly effective strategy for developing advanced composites, the potential of aerogels to be magnetically captured, recyclable, and have improved adsorption capacity, enabling efficient removal and recovery, the complexity of water pollutants still demands the design and development of multifunctional absorbents to overcome the remaining challenges.

### 2.4. Hybrid and Multifunctional Aerogel Designs

Due to the complex mix of pollutants in water, there is a need for multifunctional materials that simultaneously remove oils, pollutants, and microorganisms, which is essential for efficient treatment. In this manner, multifunctional aerogels can provide an effective alternative due to their mechanical resilience, stability, and environmental performance [92,98]. Moreover, it was observed that materials such as carbon, cellulose, polyimide, or alginate are considered to be excellent in applications involving oil-water separation, high adsorption capacity, and effective removal of heavy metals, while silk nanofibril and silver–alginate aerogels can provide antimicrobial activity [98]. Although their integrity remains a limitation, hybrid organic–inorganic and purely organic aerogels have been developed to address this, combining rigidity and elasticity to improve durability, flexibility, and environmental stability. These advancements highlight the growing potential of aerogels as sustainable, high-performance materials for ecological purification and multifunctional applications [99]. In this regard, biomass-derived materials are rich in reactive groups and can serve as versatile platforms for developing aerogels with tailored adsorption and catalytic properties [100].

Recent studies on aerogels have led to the diversification of hybrid aerogels, as presented in Table 2. Thus, the progress made in developing porous, multifunctional materials for water purification is highlighted. It can also be seen that each study focuses on evaluating different materials, from GO-based hybrids to cellulose-derived structures, MOFs, carbon nanotubes, or boron nitride-magnetic assemblies. At the same time, the key features of the materials presented are evident: high porosity, surface chemistry, and mechanical stability, which all lead to a significant improvement in pollutant absorption. Studies show that these materials can absorb a wide range of pollutants through synergistic mechanisms. In addition, the materials presented also showed reusability, structural integrity after multiple cycles of use, and scalable manufacturing. Overall, this table demonstrates the versatility of the materials and the efficiency of these architectures in developing advanced water-treatment platforms.

Based on the comparative analysis presented in Table 2 and Table 3, clear trends emerge: carbon-based aerogels dominate in the removal of dyes and pharmaceuticals via π–π interactions. In contrast, magnetic and hybrid aerogels exhibit superior performance toward metal ions through chemisorption and redox-driven mechanisms. The pollutant class, therefore, dictates both the optimal aerogel composition and the dominant removal pathway, underscoring the need for mechanism-informed material design.

**Table 3:** Multifunctional aerogels for water depollution.

Aerogel Composition	Key Features	Pollutants Removed	Mechanisms	Performance Highlights	Ref.
Graphene oxide (GO) with graphene nanoplatelets (GNPs) based aerogel	Macro- and mesoporous structure	Caffeine (CAF), Ofloxacin (OFLOX), Rhodamine B (RhB), Benzophenone-3 (BP3), Benzophenone-4 (BP4), Carbamazepine (CBZ), Bisphenol A (BPA), Diclofenac (DCF)	Hydrogen bonds between molecular functionalities and GO/rGO groups, Hydrophobic interactions, and strong π–π stacking with graphitic areas/GNP	Stable performance upon reuse, without graphene release	[101]
GO-Doped Silica Aerogel (GO-SA)	Predominantly mesoporous with slit-like, non-uniform pores, High thermal stability, and stable surface chemistry	Acid Green 25 (AG), Crystal Violet (CV), Sulfamethoxazole (SMA)	Electrostatic interactions, Hydrophobic interactions, π–π interactions	Near-complete removal Excellent reusability Removal is maintained after multiple cycles	[102]
Cellulose/Lignin/Montmorillonite Ternary Hybrid Aerogel	Hierarchically porous 3D network Macroporous architecture	Tetracycline-class antibiotics Other pharmaceuticals Organic dyes	Electrostatic interactions, Hydrogen bonding, π–π interactions, Cation-exchange	Higher adsorption affinity than pure cellulose aerogels Suitable for repeated use and flow-through applications, Good structural stability and environmentally friendly	[103]
Alginate/Silica Hybrid Aerogel Beads	Mesoporous nano-filamentous structure	Heavy metals	Surface complexation/chelation Electrostatic attraction Ion exchange/interaction with alginate carboxylates	Excellent mechanical stability Hierarchical porosity -> excellent adsorption of low concentration of Pb^2+^ Surface modification improved adsorption Environmentally friendly production	[104]
Amyloid/ZIF-8 hybrid aerogel	Ultralight, porous, mechanically reinforced High chemical robustness Hydrophobic surface	Heavy metal ions Ag^+^, Au^3+^, Hg^2+^, Cr^6+^, Cu^2+^, Co^2+^, Ni^2+^, Pb^2+^, Pt^4+^ Synthetic dyes: Acid fuchsin (degraded catalytically), Congo red, Crystal violet, Methylene blue, Malachite green, Rhodamine B Organic solvents/oils: n-hexane, acetone, cyclohexane, toluene	Chelation Electrostatic interactions π–π interactions, Hydrophobic interactions, Catalytic degradation	Ultralight and cheap precursors for its synthesis Chemical durability Scalable fabrication A universal adsorbent	[105]
Hybrid carbon aerogel, based on GO and Graphene nanoribbons (GNRs)	Ultra-low density High porosity High mechanical properties Hybrid pore walls Large pore volume	Environmental cleanup of oils and organic solvents	Hydrophobic interactions π–π interactions Rapid adsorption due to ultra-open 3D network	Maintain capacity without structural collapse Scalable fabrication Electrochemical performance	[106]
Carbon nanotube–bonded graphene hybrid aerogel CNTs grown directly from NiCl_2_ salt distributed on graphene CNTs grown from pre-reduced NiNPs	Mesoporous structure High surface area Increased electrical conductivity Magnetic behavior due to NiNPs	Dyes: Methylene Blue, Crystal Violet, Congo Red, Methyl Orange Promote the simultaneous removal of anionic and cationic dyes (e.g., methylene blue, methyl orange) Selective absorption of toluene, oils, and organic solvents from water	π–π interactions Van der Waals forces Mass transfer due to the aerogel’s high porosity Magnetic separation	Promote simultaneous adsorption Reusability Promote rapid and selective adsorption of organic solvents Maintains structural integrity	[107]
Hexagonal boron nitride/PEI/magnetite (MHA) hybrid	Polygonal mesoporous structure Rich functional groups such as –NH_2_, –NH, –N, –OH, B–N, B–O Magnetic properties	Heavy metals: Cr(VI), As(V) Organic dyes: methylene blue, acid orange	Electrostatic attraction Hydrogen bonding Multilayer adsorption Redox reactions	Reusability Regeneration Magnetic separation	[108]
Magnetic mesoporous iron–carbon aerogel (Fe/CA)	High surface area and mesoporosity Strong ferromagnetic behavior enabling magnetic separation	As(V) ions	Arsenic binding to iron active sites within the mesoporous carbon matrix	Rapid magnetic recovery without centrifugation	[109]
Graphene–iron nanoparticle aerogels (graphene–αFeOOH and graphene–Fe_3_O_4_)	3D graphene network decorated with iron nanoparticles	PO_4_^3−^	Pseudo-second-order kinetics (chemisorption) Formation of mono- and polynuclear surface complexes on iron sites	High efficiency at elevated phosphate concentrations Magnetic recovery Fast kinetics	[110]
Graphene aerogel/cellulose fibers/magnetite nanoparticles (GCM) composite	3D graphene aerogel network, reinforced with cellulose fibers, incorporating Fe_3_O_4_ NPs Mesoporous structure Hydrophilic surface Magnetic behavior	Gold cyanide complex Au(CN)_2_^−^	π–π interactions between graphene sheets and Au(CN)_2_^−^ complexes	Easily recovered via magnetic separation Retains structural integrity after multiple utilizations	[111]
Magnetic mesoporous Fe_3_C/carbon aerogel	Carbon-based aerogel framework Mesoporous structure Strong magnetic response	As(V)	Chemisorption-dominated adsorption Surface complexation/Ligand exchange between arsenate	Magnetic separation Fast uptake	[112]

## 3. Ecotoxicological and Environmental Safety Evaluation

The evaluation of the ecotoxicological and environmental safety of emerging aerogels requires a precise understanding of how their intrinsic physicochemical properties influence interactions with living and non-living systems. In this regard, the toxicity of aerogel-based materials can be influenced by the solvents used in the synthesis route [113]. Moreover, the nature of the polymer can affect the properties of aerogels, including mechanical strength, functionality, and stability [114].

Thus, aerogels fabricated from natural polymers (e.g., cellulose, chitosan, alginate, starch) present good biocompatibility and environmental sustainability [114,115]. However, studies have provided insights into their potential mild to moderate toxicity, which can depend on the formulation and exposure conditions [115]. Also, synthetic or carbon-based systems have been reported to cause problems such as DNA damage, pulmonary toxicity, mesothelioma, inflammation, genotoxicity, neurotoxicity, and adverse effects on human embryo development [115,116]. Additionally, incorporating functional additives into bioaerogels, such as polyethyleneimine, polyaniline, graphene oxide, and surfactants (e.g., sodium dodecyl sulfate), can enhance aerogel adsorptive properties. Still, it can increase aquatic and cellular toxicity, linked to genotoxic and teratogenic effects in aquatic species, including Zebrafish and marine copepods, if the aerogel is poorly made or recycled [117]. For example, in the context of silica-based aerogel fabrication, studies reported the presence of silica NPs in surface water and oils, and their effects on microorganisms and plants, such as *Paracentrotus lividus* embryos, involve developmental abnormalities at higher nanoparticle concentrations [118]. Casado et al. [119] reported that aquatic organisms such as Daphnia magna exposed to silica NPs did not exhibit toxicity up to >1000 µg mL^−1^. Compared to the Casado et al. study, the research by Clément et al. [120] on *Daphnia magna* exposed to commercial silica NPs showed acute toxicity (EC_50_ = 29.7 ± 1.5 mg L^−1^), higher than that observed in other species tested. Moreover, the literature reports that silica-based aerogels can exhibit higher bioactivity than polymeric ones, as observed with alveolar macrophages, which induce TNF-α at ≥90 µg mL^−1^. However, these nanostructures can be internalized, and the ultrafine fragments may interact with biological tissues through oxidative mechanisms, which is an early and reliable indicator of nanomaterial reactivity [121]. In contrast, polymer-based aerogels have low oxidative potential and, as observed on alveolar macrophages, a low inflammatory response. Moreover, chitosan and carbon-based aerogels exhibit mild redox activity due to their surface chemistry or residual synthesis components [121,122]. Due to the frequent functionalization of aerogels with metal or metal oxide NPs, it is crucial to consider their individual toxicity profiles for their safe use in water decontamination. In this regard, aerogels’ toxicity can be attributed to NPs leaching and the potential to induce oxidative stress [123]. In aquatic environments, NPS can accumulate, forming aggregates and sediments, and can interact with marine life and plants [124]. In an aquatic environment, invertebrates such as *Daphnia magna*, copepods, and protozoans are prone to ingest NPs, while the Zebrafish can absorb them through the chorion [125]. On the other hand, metal and metal oxide NPs have the potential to alter the behavior and cause mortality in aquatic species, such as Zebrafish and Daphnia [126]. Moreover, they tend to accumulate in the guts of Daphnia or can cause gill lesions, while the chronic exposure triggers the oxidative damage and immune alterations in Zebrafish [124,125,126].

These effects were also observed in MNPs. As mentioned earlier, MNPs’ toxicity in Zebrafish models demonstrates that MNPs can cause histopathological changes in organs such as the gills and intestines, as well as neurobehavioral disturbances [127]. These findings were confirmed by studies, such as that by Kaloyanni et al. [128], which investigated the toxic effects of Fe_3_O_4_ in animal models representing both terrestrial (*Cornu aspersum* snails) and aquatic ecosystems (Zebrafish and Prussian carp). Following Fe_3_O_4_ exposure, the studied species exhibited significant increases in lipid peroxidation and protein oxidation, accompanied by increases in markers of apoptosis and protein degradation at the following concentrations for Cornu aspersum: 12.5 µg mL^−1^, Zebrafish liver: ~1 mg L^−1^, and Prussian carp: 300 mg g^−1^. In contrast, DNA damage increased for all organisms. Additionally, Zhu et al. [129] demonstrated the toxic potential of α-Fe_2_O_3_ against the Zebrafish embryo. In their study, α-Fe_2_O_3_ caused embryonic malformations and mortality. Moreover, the researchers suggested that the toxicity is primarily linked to NPs’ tendency to aggregate, direct surface interactions, and the possible release of iron, which can lead to oxidative stress and developmental disruption. Blinova et al. [130] observed that a short exposure to Fe_3_O_4_ caused little immediate mortality in *Daphnia magna*; however, long-term effects were observed after the daphnids were transferred to clean water. Concentrations of 10 or 100 ppm of MNPs increased mortality, while surviving individuals showed increased reproductive output.

**Figure 5:**
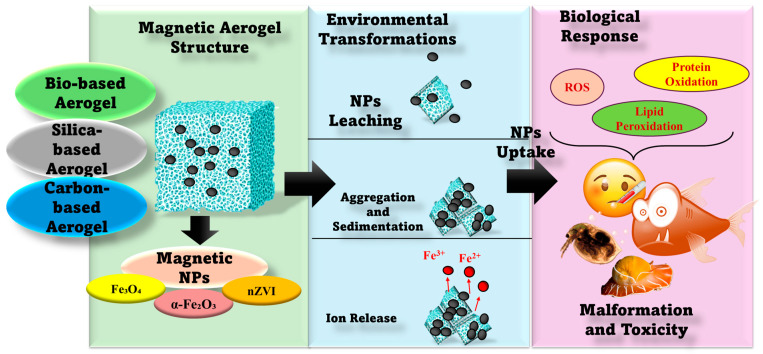
Schematic overview of structure–toxicity relationships in aerogel-based magnetic nanocomposites.

To better understand the reported ecotoxicological data, Figure 5 schematically summarizes the effects of magnetic aerogel structural features, NPs-associated environmental impact, and the resulting biological responses. The figure provides insights into how the aerogel matrix composition, magnetic NPs stabilization, and surface modification can impair NPs leaching, aggregation, and iron release, thereby preventing oxidative stress pathways and toxicity outcomes in representative aquatic and terrestrial models.

Based on reported effect thresholds and mechanistic responses, it can be concluded that polymeric and bio-based aerogels are less toxic than inorganic aerogels. Moreover, the inorganic aerogels need improvement in their biocompatibility. Silica-based materials are still considered moderately toxic, while metal and metal oxide materials remain a concern for ecotoxicology. Based on the studies presented, oxidative stress, DNA damage, and inflammatory responses can serve as early warning indicators of toxicity induced by aerogel-based materials, which are attributed to NPs that can cause oxidative stress, lead to cell damage, trigger inflammatory reactions, and cause adverse effects due to their leakage. These effects can have a significant impact on aquatic organisms if the materials are not properly stabilized or recycled, rendering them ineffective for their intended purpose. However, further studies are needed to better understand the toxicity potential of aerogels and assess their safe use in real-world applications.

## 4. Regeneration, Recyclability, and Operational Stability

### 4.1. Intrinsic Material Stability and Regeneration Performance

Magnetic recovery of aerogels can be achieved through the magnetic properties of the iron oxide NPs, especially Fe_3_O_4_, which enable rapid separation, recovery, and reutilization with external magnets [66,76,131,132]. In this sense, by incorporating MNPs into materials, it is possible to obtain composites with recovery potential, providing them with strong magnetization (saturation magnetization between 20–100 emu g^−1^) that minimizes secondary pollution and enables continuous operation [43,133,134]. Due to their superparamagnetic or ferrimagnetic properties, magnetic manipulation prevents agglomeration, ensuring rapid retrieval from solution while maintaining dispersion stability. The magnet strength, particle dispersion, and composite structure influence the magnetic recovery process. Moreover, compared to individual NPs, which require a strong magnetic field, material composites such as aerogels exhibit enhanced responsiveness [76]. Thus, such materials are used in water depollution, providing a fast, low-cost, and recyclable alternative to conventional methods (e.g., centrifugation, flotation, or biological treatment), which otherwise provide a poor separation efficiency and can contribute to secondary pollution [132,135,136].

In addition to rapid magnetic recovery and separation, aerogels can offer high recyclability and operational durability, sustaining performance after multiple regeneration cycles. Such adsorbents are considered critical determinants of practical feasibility [133]. In this regard, several studies provide insights into the operational stability and recyclability of aerogels, which are presented in Table 4. Thus, the table aims to highlight the regeneration performance of aerogels reported in the literature, including reuse cycles, capacity retention, and regeneration methods. It can be seen that magnetic or carbon-based aerogels exhibit superior recyclability (80–90%) over multiple cycles of use. This may be due to their robust structure, as well as to regeneration strategies. On the other hand, silica- or cellulose-based aerogels exhibit moderate to low reuse, possibly due to structural degradation during regeneration.

**Table 4:** Regeneration performance of various aerogels used as adsorbents.

Adsorbent Type	Cycles	Capacity Retained	Regeneration Method	Ref.
Magnetic MnFe_2_O_4_–cellulose aerogel	Multiple cycles	>80%	Ethylenediaminetetraacetic acid (EDTA)	[48]
Hydrophilic silica aerogel (HPSA)	3	80%	Thermal Regeneration	[50]
Titania-doped silica aerogel (TdS)	4	MB: 84% CV: 80%	Ethanol Washing	[51]
GO-Doped Silica Aerogel (GO-SA)	5	85%	Selective Chemical Washing + Solvents	[102]
Hybrid carbon aerogel, based on GO and Graphene nanoribbons (GNRs)	10	90%	Thermal Regeneration	[106]
Cellulose-based aerogel	10	45%	Mechanical Pressing	[137]
Magnetically responsive carbon-based aerogel system	5	71%	Hexane Washing + Drying at 80 °C	[138]
Superhydrophobic, magnetic nanocellulose-based aerogel	5–10	50%	Mechanical Pressing	[63]
		90%	Ethanol Washing	

**Figure 6:**
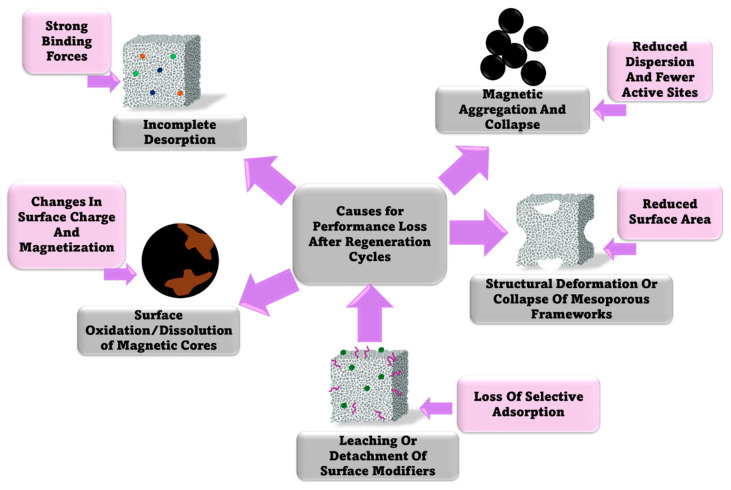
Causes of performance loss after multiple regeneration cycles. Based on information from [133]. Colored spheres represent different pollutants adsorbed by magnetic aerogels, while black spheres indicate MNPs.

However, as observed, aerogels begin to lose performance after multiple regeneration cycles, which is caused by several physicochemical factors (Figure 6) and can affect the economic sustainability and environmental impact of magnetic nanoadsorbent-based systems [133]. The progressive loss of the adsorptive properties due to repeated regeneration cycles is often associated with incomplete desorption of strongly bound pollutants, a principal factor that contributes to performance loss, degradation of adsorbent structure, and leaching or loss of surface functional groups, pore blockage, which results in limited available active sites, and promotes aggregate formation after successive cycles [139]. In the case of magnetic materials, factors such as oxidation, dissolution, and passivation of the magnetic core, with their leaching and instability, are considered. These cumulative factors can lead to decreased regeneration and inefficient long-term use of materials, due to their high tendency to aggregate and low dispersibility [140]. Repeated regeneration can also promote pore collapse, surface redox reactions, and aggregation-induced loss of dispersion stability, collectively hindering access to adsorption sites and accelerating capacity decay over multiple cycles [141].

However, the regeneration process for magnetic aerogels represents an important step, ensuring the technological and economic sustainability of adsorption-based systems. This process enables the multiple utilization of aerogels with minimal loss of performance. The selection of a suitable regeneration method depends largely on the cost, chemical stability, and nature of the adsorbent material [142]. The most well-known techniques for aerogel regeneration include chemical, thermal, solvent extraction, and microbial regeneration [133]. Chemical regeneration is considered to be highly effective regarding magnetic nanoadsorbents, such as aerogels. For example, NaOH can restore the adsorption activity, providing high reusability capacity and stability [143]. Likewise, the use of dilute HNO_3_ for chemical regeneration enhances metal desorption and maintains the adsorbent’s integrity and performance across multiple cycles [144]. In contrast, thermal regeneration offers a cleaner, more sustainable alternative to aerogel regeneration, enabling complete removal of pollutants. However, this method still needs improvement in thermal conditions, monitoring emissions, and ensuring adsorbent stability to enable safe, repeated use [145]. Combining two methods effectively preserves pore structure and adsorption capacity, demonstrating the improvement regarding their durability over 17 cycles. Still, the high time and energy requirements can limit industrial applications [146].

Even though aerogels can be regenerated, they still have drawbacks that have not advanced them beyond the laboratory stage. In this regard, magnetic aerogels still suffer from incomplete desorption, surface oxidation, and the risk of structural collapse or aggregation [133].

### 4.2. Environmental Adaptibility in Complex Aqueous Systems

For real-world applications, the long-term stability of aerogels in aqueous environments is a key factor in determining whether these materials can advance from the laboratory stage to practical water treatment and remediation strategies. Several studies have demonstrated that the chemical network of aerogels, rather than their porosity, determines their durability under realistic conditions.

Thus, Paraskevopoulou et al. [147] developed a polyurea-crosslinked calcium alginate aerogel as an advanced porous sorbent for seawater decontamination. This material was investigated for the adsorption of heavy metal ions, with a particular focus on lead (Pb^2+^). The explosion of multi-component metal solutions, the aerogel presented a strong and selective affinity for lead, with an adsorption capacity of about 29 mg g^−1^ in ultrapure water. In contrast, in seawater it remained at about 13 mg g^−1^. In ultrapure water, the adsorption followed a Langmuir isotherm, indicating adsorption on uniform sites, whereas in seawater, the adsorption followed a Freundlich isotherm, suggesting heterogeneous adsorption due to competition among dissolved ions. Moreover, researchers demonstrated that the aerogel can be regenerated without damaging the aerogel framework. After regeneration, the aerogel exhibited adsorptive properties nearly as efficient as before over multiple cycles.

A related study was performed by Georgiou et al. [148]. The researchers designed a polyurea-crosslinked alginate aerogel platform for uranium removal and recovery from aqueous environments. The aerogel was tested in acid mine drainage, groundwater, and uranium-spiked seawater, demonstrating that its structure remains intact, with no swelling, shrinking, or disintegration, and that it retains regenerative capacity. In this sense, this aerogel exhibited fast kinetics and long-term stability in different aqueous environments, making it suitable for radioactive water decontamination and uranium recovery.

Another complementary design was proposed by Qiang et al. [149]. The researchers created amidoxime-functionalized collagen/alginate aerogel beads, aiming to extract uranium from seawater. This aerogel provides high adsorption capacity with antibacterial functionality. Moreover, the study addresses the biofouling problem and demonstrates aerogel’s long-term stability in coastal seawater over 30 days, maintaining its adsorptive properties and mechanical integrity.

Another study on the long-term stability of aerogels was performed by Fu et al. [150]. The researchers proposed a cellulose-based aerogel for oil–water separation. This aerogel demonstrated high efficiency in water-oil separation and robust durability after several reutilizations in acids, bases, and high-salinity environments, making it a suitable material for harsh aqueous environments.

In real aqueous environments, the performance of magnetic aerogels can be influenced by their magnetic behavior, competing species, and matrix complexity. Magnetic separation offers advantages, such as a magnetically controllable platform for selective adsorption, owing to magnetic properties that are key determinants of adsorption capacity and separation performance [151,152]. Thus, incorporating magnetic particles can contribute to greater capture and improved recovery by generating stronger magnetic fields at higher saturation [152]. In adsorbent systems, capture efficiency can also be determined by the penetration time and saturation capacity, which depend on the balance among magnetic force, particle magnetization, and hydrodynamic resistance. In this case, the recyclability and short-term stability of these materials are achievable, as repeated cycles do not cause significant losses in magnetism. Still, their long-term storage may lead to substantial performance degradation due to oxidation [153,154].

About aerogel-based adsorbents, competitive adsorption, ionic strength, and natural organic matter (NOM) can influence the adsorption capacity of the material. Thus, in environments with multiple contaminants, coexisting ions compete for active sites, while ionic strength filters electrostatic interactions, which, over time, reduces the adsorption efficiency of aerogels. NOM can also contribute to pore blockage, hindering or preventing adsorption [155]. In this regard, selecting an optimal composition during aerogel manufacture can significantly mitigate these limitations. An example of this is Nie et al. [156], who designed a citrus peel/chitosan/bentonite-based aerogel that demonstrated high selectivity and adsorption in liquid media containing mixed metals, high ionic strength, and NOM, due to dominant chemical chelation mechanisms rather than purely electrostatic binding. Similarly, Yu et al. [157] proposed a lanthanum-modified biopolymer aerogel that exhibited improved resistance to competitive sorption and NOM, as evidenced by ciprofloxacin adsorption capacity testing.

Overall, the findings suggest that aerogels can exhibit long-term stability in aqueous environments, which depends on their robustness and chemical structure. The possibility of use in harsh aqueous environments was demonstrated, with mechanical integrity and functional performance maintained.

## 5. Scale-Up Challenges and Future Perspectives

Despite promising progress reported in the literature on magnetic aerogels, several contradictions and limitations remain and need to be addressed. It has been observed that, from one aerogel to another, the adsorption capacity varies widely for similar pollutants. The experimental conditions are the main factors determining the differences in this regard, as are the surface functionalization strategies and the kinetic and isothermal models used. In addition, regeneration efficiency and long-term operational stability are assessed inconsistently, with most studies limited to a small number of reuse cycles under idealized laboratory conditions. These inconsistencies prevent effective comparison between studies and highlight the need for standardized testing protocols so that results can be interpreted more critically and the performance and use of these materials in real applications can be improved.

In this sense, the aerogels are constantly evolving and have potential for various applications, but still pose challenges that must be overcome to transition from the laboratory to practical use [158]. Aerogel’s long-term stability is a primary concern regarding its application. Their structure is inherently fragile and has poor mechanical properties, which makes handling and processing difficult [21,159]. Moreover, the regeneration and reuse of aerogels remain challenging due to their strong chemisorptive interactions with adsorbed pollutants, which hinder effective desorption. In this case, the adsorbed pollutants irreversibly remain in the aerogel’s network [10]. Chemical buffers improve desorption, but they also generate secondary waste, increase operating costs, and pose additional environmental risks. Therefore, further studies are needed to initiate improved strategies for ecological regeneration and the reduction in secondary pollution [10,25].

Laboratory-scale synthesis routes such as sol–gel, supercritical drying, and freeze-drying tend to be slow, energy-intensive, and equipment-dependent, which can increase time, slow down production, increase costs, and limit throughput [158,159,160]. Moreover, the transition to industrial scale should require two key shifts: a change to semi-continuous or continuous manufacturing, the replacement of gradual solvent exchange with a flow-optimized one, and drying protocols that fluidize the process, reduce cycle times, and minimize solvent waste [25,161].

Given the limitations of manufacturing aerogels, interest has shifted to modern processing approaches that can overcome some of the drawbacks of conventional synthesis, particularly related to geometry and scalability. Additive manufacturing (AM) or 3D printing is utilized to enhance the properties of aerogels, including high porosity, thermal insulation, and stability, thereby enabling the development of a new generation of aerogels due to its high precision [162,163,164,165]. Aerogel synthesis through AM is achieved using a nozzle aimed at forming stable filaments. This technique promotes structural customization and directional freezing, which helps align pores, and post-treatments, such as thermal reduction or sintering, to improve the strength of the aerogel [162]. However, aerogel inks used in AM remain challenging due to their lack of shear-thinning, yield-stress, and thixotropic behavior. These drawbacks are overcome by incorporating 1D, 2D, or composite additives; however, this approach decreases the purity, homogeneity, and aerogel properties [166,167]. Even if these additives are used, the printing is still limited. The aerogel inks have low solid contents, a time-dependent sol–gel transition, and a high tendency to obtain clogged or structurally unstable products within minutes, undermining reproducibility. In this sense, complex geometries are challenging to achieve, as the aerogels are too fragile due to low-modulus inks that sag, spread, or collapse, while stiffer formulations fracture or clog [168]. Moreover, due to inefficient mixing and poor reproducibility in sol–gel synthesis of aerogels, researchers have suggested an alternative, represented by microfluidic synthesis, a promising strategy for producing uniform, controllable, and scalable structures [169,170]. In this regard, microfluidic platforms can enhance the porosity of aerogel materials at the micrometer scale and, upon solvent removal, generate a porous network of interconnected pores [19]. Additionally, integrating microfluidic platforms into magnetic aerogel synthesis can control the incorporation of MNPs into the aerogel structure [171,172]. After synthesis, aerogels are dried by supercritical CO_2_ drying, freeze-drying, or ambient drying; the choice is made to minimize structural collapse [163].

Drying remains a crucial step in aerogel fabrication, as it can significantly impact scalability and industrial implementation. Ambient pressure drying (APD) is a less expensive technique than supercritical drying (SCD), replacing its high pressure and temperature with a simpler, lower-cost process performed at or near atmospheric pressure [173,174]. However, the aerogel structure can collapse due to capillary forces that arise as the liquid begins to evaporate [175]. A strategy for improving the aerogel structure can be achieved by changing the polar solvents (e.g., ethanol, water) used during synthesis or by surface modification with hydrophobic groups [174]. In contrast, freeze drying (FD) is a simple, more eco-friendly alternative to the aerogel’s manufacturing process. Thus, this process involves sublimation of solvents from the gel pores under vacuum, thereby avoiding the surface tension associated with the gas–liquid phase transition [176,177]. However, the FD process still faces limitations regarding the aerogel synthesis, including structural damage, uncontrollable pore size, and low freezing efficiency [177,178]. One of the main challenges is optimizing the phase transition stage during the freezing step, such as directional freezing, which can help achieve precise structural control. As a result, the development of controlled-freezing methods can improve the aerogel’s structure and performance [177]. These methods promise to preserve hierarchical porosity while ensuring compatibility with conventional processing units [160]. Thus, even if the APD and FD represent a cheaper alternative, they require complex pretreatments, which result in longer production times. At the same time, FD has an increased tendency to damage the porous structure through ice crystal formation [159,178]. However, SCD remains the primary strategy for providing a high-quality, low-density structure with fine porosity, enabling more scalable aerogel production [179].

Nevertheless, magnetic aerogel fields are still in development, moving beyond traditional inorganic silica aerogels to organic and bio-derived aerogels, which can enhance materials’ properties, such as biocompatibility, elasticity, biodegradability, and tunable adsorption chemistry [161]. Moreover, these materials can address sustainability concerns by using renewable resources such as cellulose, chitosan, and alginate [180,181]. However, the use of these materials for large-scale aerogel production is limited by their poor mechanical properties, low thermal stability, and drying challenges [181]. Additionally, natural polymers require harsh chemical treatments during processing. Therefore, research must focus on finding solutions and implementing green chemistry, which offers safer methods with reduced toxicity for oxidation, bleaching, and extraction [180].

Another strategy in water decontamination is represented by the development of “smart” or stimuli-responsive aerogels. These aerogels have the capacity to adjust their physicochemical properties in response to environmental stimuli [182,183,184]. For example, smart aerogels, known as 4D aerogels, exhibit adaptive regeneration and self-healing functionality [184,185]. Thus, titanium-organic aerogel can promote dye degradation through the photo-induced generation of ROS, due to its reversible reduction of Ti^4+^ to Ti^3+^ under UV irradiation and subsequent air exposure oxidation [183]. Similarly, a bio-inspired, multiple-stimuli-responsive graphene oxide/poly(vinyl alcohol) aerogel, developed by Sun et al. [185], can reversibly change its shape in response to external factors such as humidity and polarity, with effects that can be programmed for transformation and shape memory.

However, these materials are still in the research stage due to several challenges that continue to hinder their practical application in real-world water purification systems. In this sense, to enhance the pollutant binding and desorption kinetics, advanced characterization methods and modeling approaches are needed [10]. For instance, integrating computational tools, such as machine learning (ML) and predictive modeling (PM), can help optimize aerogels’ performance. ML algorithms (e.g., Support Vector Machines, Random Forests, Artificial Neural Networks, and Gradient Boosting) can predict water quality parameters, identify pollutant types, and optimize aerogel composition and structural features, compared to traditional modeling approaches [186,187]. Moreover, in material design, these strategies demonstrated that algorithms can identify correlations between synthesis parameters and functional properties, thereby reducing the need for trial-and-error experimentation [188]. In this regard, the implementation of Random Forest and Gradient Boosting models demonstrated high predictive power for the aerogels’ structural features and their adsorption capacities for pollutants, such as dyes and heavy metals [189]. In the same manner, data-driven models were used to predict adsorption of metal–organic frameworks for antibiotics and perfluoroalkylated acids, providing insights into the interactions between material structure and pollutant characteristics [190].

Another important factor in the development of aerogels is their economic and ecological impact, and this depends not only on the choice of raw materials but also on the manufacturing pathways. For example, conventional manufacturing production can represent an environmental burden due to volatile organic compound (VOC) emissions. This can be avoided through greener approaches, such as replacing with supercritical CO_2_, or shifting toward solvent-minimized and solvent-free processes. This can reduce the overall energy demand [191,192]. Thus, aerogel’s future depends on its capacity to reduce costs and its environmental footprint simultaneously. For instance, introducing materials such as silicon derived from waste (e.g., rice husk ash and recycled glass) or bio-based polymer precursors can considerably reduce raw-material costs, enabling industrial and agricultural waste to be transformed into high-value adsorbent materials [136,191,192]. In addition, other strategies may involve transitioning from high-energy supercritical drying to APD methods, and AM can enable continuous mass production of aerogels with tailored geometry, function, and performance, ready for use in applications such as environmental remediation [162,191].

To develop a new research perspective on the challenges and opportunities for improving the performance of magnetic aerogels, two complementary research frameworks are proposed.

**Figure 7:**
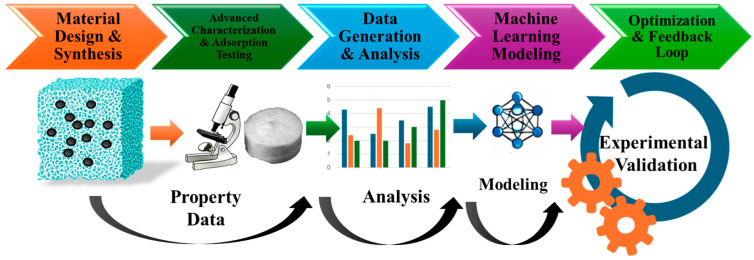
Data-driven framework for smart aerogel optimization.

In this regard, the first framework is based on data-driven design and the optimization of smart aerogels. Thus, this framework demonstrates the feasibility of integrating material design, advanced characterization, and data-driven modeling to enable the development of stimuli-responsive aerogels. The proposed roadmap is presented in Figure 7. This strategy can limit trial errors in experiments, enabling predictive optimization of aerogel properties and their applicability to a specific class of pollutant removal.

**Figure 8:**
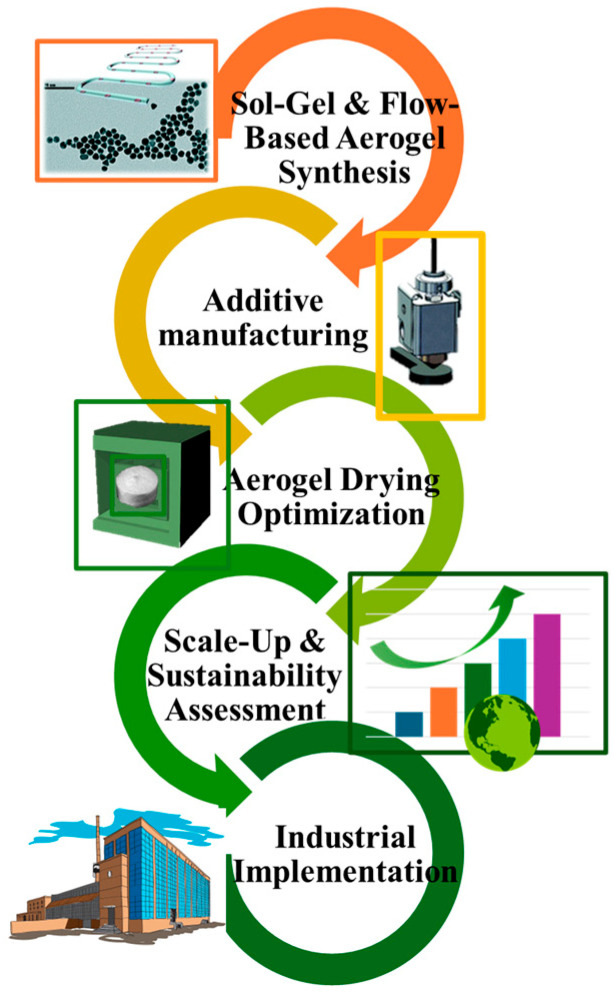
Scalable manufacturing roadmap for aerogels optimization.

The second proposed framework aims to overcome scalability and reproducibility limitations in the synthesis of aerogels through advanced manufacturing strategies. In this regard, the proposed roadmap is presented in Figure 8. Together, these steps provide a structured pathway toward large-scale, sustainable production of aerogels for environmental remediation.

Overall, these insights underscore the potential to extend aerogel technologies by integrating advanced manufacturing methods, selecting sustainable materials, and adopting data-driven optimization strategies, enabling the transition from promising laboratory concepts to reliable, large-scale solutions for environmental remediation.

## 6. Conclusions

Given the burden of the global water pollution crisis, there is a need to develop and implement innovative, effective strategies to depollute water. In this respect, magnetic aerogels can serve as an advanced strategy for water decontamination due to their high porosity, surface chemistry, and multifunctionality. The integration of magnetic NPs can enhance pollutant adsorption, catalytic degradation, and rapid magnetic separation, due to their magnetic properties. Thus, this can enable the efficient removal of pollutants, such as heavy metals, dyes, pharmaceuticals, and persistent organic compounds. Moreover, the implementation of hybrid aerogels can enhance their reusability, structural integrity after multiple use cycles, and scalable manufacturing.

However, some challenges need to be overcome. There is still a risk of NPs leakage, which produces ROS and remains a concern for ecotoxicity, as well as the structural fragility of aerogels, incomplete desorption, and oxidation of the magnetic core. Furthermore, aerogel optimization can be achieved by improving material stability, optimizing synthesis methods, implementing environmentally friendly strategies, and adopting advanced manufacturing techniques. In addition, the integration of machine learning and intelligent architectures is promising for optimizing design and enabling adaptive pollutant removal.

Overall, aerogel materials have significant potential for water depollution, but their transition from laboratory research to large-scale industrial applications still needs optimization and ongoing interdisciplinary research.

## Data Availability

No new data were created or analyzed in this study. Data sharing is not applicable to this article.
